# Supplementary figures and images for: Clinical significance of C4d deposition in renal tissues from patients with primary Sjögren’s syndrome—a preliminary study
Source: BMC Nephrol. 2019 May 28;20:189. doi: 10.1186/s12882-019-1341-y (PMC6540533; doi:10.1186/s12882-019-1341-y)

Figures


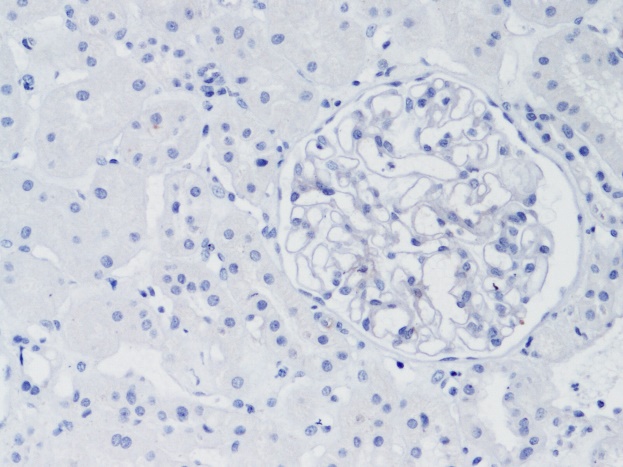

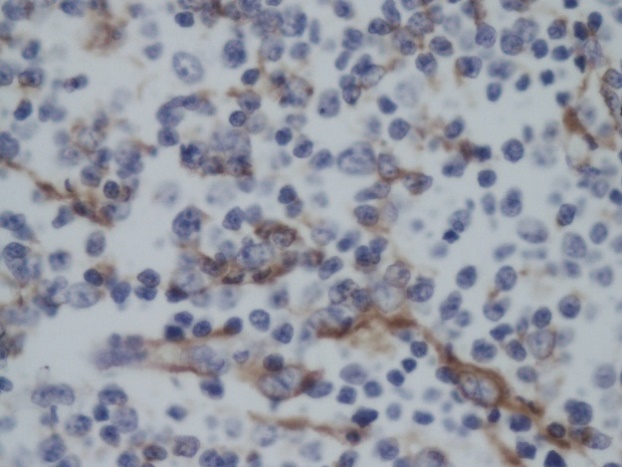


**S1**

**S2**

Supplement: Supplementary file 1 — Figure S1. × 200, positive control, C4d stained dendritic cells in the tonsil tissues. Figure S2. × 200, negative control, renal biopsy tissues from a patient with minor lesions. (DOCX 604 kb) [file 12882_2019_1341_MOESM1_ESM.docx]
